# Seed osmopriming with polyethylene glycol (PEG) enhances seed germination and seedling physiological traits of *Coronilla varia* L. under water stress

**DOI:** 10.1371/journal.pone.0303145

**Published:** 2024-05-10

**Authors:** Leyuan Ma, Jingui Wei, Guojun Han, Xiaomei Sun, Xiaobing Yang

**Affiliations:** 1 College of Resources and Environmental Sciences, Gansu Agricultural University, Lanzhou, Gansu province, China; 2 College of Agronomy, Gansu Agricultural University, Lanzhou, Gansu province, China; Hainan University, CHINA

## Abstract

Water stress can adversely affect seed germination and plant growth. Seed osmopriming is a pre-sowing treatment in which seeds are soaked in osmotic solutions to undergo the first stage of germination prior to radicle protrusion. Seed osmopriming enhances germination performance under stressful environmental conditions, making it an effective method to improve plant resistance and yield. This study analyzed the effect of seed osmopriming with polyethylene glycol (PEG) on seed germination and physiological parameters of *Coronilla varia* L. Priming treatments using 10% to 30% PEG enhanced germination percentage, germination vigor, germination index, vitality index, and seedling mass and reduced the time to reach 50% germination (T50). The PEG concentration that led to better results was 10%. The content of soluble proteins (SP), proline (Pro), soluble sugars (SS), and malondialdehyde (MDA) in *Coronilla varia* L. seedlings increased with the severity of water stress. In addition, under water stress, electrolyte leakage rose, and peroxidase (POD) and superoxide dismutase (SOD) activities intensified, while catalase (CAT) activity increased at mild-to-moderate water stress but declined with more severe deficiency. The 10% PEG priming significantly improved germination percentage, germination vigor, germination index, vitality index, and time to 50% germination (T50) under water stress. Across the water stress gradient here tested (8 to 12% PEG), seed priming enhanced SP content, Pro content, and SOD activity in *Coronilla varia* L. seedlings compared to the unprimed treatments. Under 10% PEG-induced water stress, primed seedlings displayed a significantly lower MDA content and electrolyte leakage than their unprimed counterparts and exhibited significantly higher CAT and POD activities. However, under 12% PEG-induced water stress, differences in electrolyte leakage, CAT activity, and POD activity between primed and unprimed treatments were not significant. These findings suggest that PEG priming enhances the osmotic regulation and antioxidant capacity of *Coronilla varia* seedlings, facilitating seed germination and seedling growth and alleviating drought stress damage, albeit with reduced efficacy under severe water deficiency.

## 1. Introduction

Plant seed germination and seedling growth are influenced by both internal physiological-biochemical regulations and external environmental factors, representing the most sensitive phase of plant growth to external stimuli [1,2]. Water deficiency poses a threat to processes such as seed germination and early seedling growth by inducing oxidative damage and metabolic disruptions in plant cells [3,4]. It is one of the most common adverse factors in plant growth environments. Osmotic regulation is one of the key physiological mechanisms enabling plants to adapt to and withstand drought and to utilize water more efficiently [5]. Plant cells metabolize different compounds and produce organic solutes such as soluble carbohydrates and amino acids. These solutes help cells regulate their own osmotic potential, which in turn enhances their ability to absorb water from the environment and maintain normal metabolism, thus mitigating the extent of cellular and tissue damage caused by water limitation [6].

Water scarcity disrupts the balance between the generation and elimination of reactive oxygen species (ROS) within plant cells. The ensuing imbalance leads to excessive ROS accumulation, lipid peroxidation, disruption of cell membrane structure and function, electrolyte leakage, and ultimately results in plant death [7]. Antioxidant enzymes play a pivotal role in plant resistance to oxidative stress. These enzymes, including superoxide dismutase (SOD), peroxidase (POD), and catalase (CAT), effectively scavenge reactive oxygen radicals when plants face adverse conditions, protecting cells from oxidative damage, thereby enhancing plant stress tolerance [8,9].

Seed priming, a pre-sowing partial hydration of seeds, is often used in agriculture and restoration to induce the early emergence of seedlings through the regulation of metabolic processes in the early phases of germination [10]. Priming essentially involves partial imbibition (as opposed to full hydration) of seeds by applying various strategies, such as shortening imbibition duration (hydropriming) or exposing seeds to relatively low external water potential (osmopriming, matric priming, or drum priming) [11,12].Treating seeds with priming agents enhances seed vigor, promotes seed germination and seedling growth [13], and improves emergence rate and seedling uniformity [14]. It has been reported that seed priming increases seed tolerance to adverse conditions like drought [15,16], salinity [17], and low temperatures [18]. However, the physiological mechanism of seed priming in relation to improved germination and stress tolerance is not fully understood.

Polyethylene glycol (PEG) is an osmotic polymer that cannot permeate seed cells. Because it is a chemically inert compound, PEG does not impose damaging effects on seed embryos. PEG priming exposes seeds to a medium with low water potential, reducing the rate of water entry into seeds and preventing visible germination [19]. Recent studies have found that PEG priming affects seed germination rate, seed vitality, and seedling development [20], promoting the germination of hard or aged seeds [21]. PEG priming not only enhanced CAT, POD, and SOD activities in plants such as *Avena sativa* L. [22], *Zea mays* L.[23], and *Oryza sativa* L.[24], thereby reducing cellular ROS levels but also decreased malondialdehyde (MDA) accumulation and cell membrane permeability in *Sorghum bicolor* L. seedlings [25]. It has been reported that PEG priming can improve plant stress tolerance by regulating the content of osmotic regulatory substances like soluble sugars (SS) and proline (Pro) [26]. In addition, PEG is non-damaging to proteins and does not penetrate seed cell membranes due to its large molecular size. Because PEG can be used to formulate solutions with specific water potentials, mimicking both normal or stressful soil water potentials, these solutions may act as osmotic stress agents for seed germination [27]. By simulating water stress, it is possible to observe seed germination capacity and physiological changes associated with plants’ drought resistance [28] and provide relevant insights into drought tolerance/resistance mechanisms, which, ultimately, may guide the timely breeding of drought-resistant and drought-tolerant varieties.

*Coronilla varia* L. is a perennial forage grass native to Europe and the Mediterranean region [29]. Introduced to China in 1973, *Coronilla varia* L. is a versatile plant resistant to drought, cold, and desertic conditions. It is also an excellent plant for soil and water conservation. Additionally, it is a highly nutritious and productive forage and green manure crop, with a good grazing tolerance, many rhizomes, and a strong ability to fix nitrogen. Besides, *Coronilla varia* L. is an excellent ornamental plant often used for highway slope protection due to its long flowering period and rapid turf formation [30]. The seeds of *Coronilla varia* L. show high hardness, low germination rates, prolonged germination periods, and uneven sprouting. This plant species primarily grows in arid and semi-arid regions such as Northwest and North China [31], with water availability limiting its widespread cultivation.

Current research on *Coronilla varia* L. primarily focuses on cultivation techniques [32,33], soil remediation [30,34], and silage [35], and there is limited data available on the mechanisms exerted by the seeds of this species to overcome water limitations and other adverse germination conditions. Particularly, studies on the physiological and biochemical changes during seed germination and seedling growth linked to osmotic seed priming are scarce, and the research on PEG use as a seed priming agent is quite new and requires further exploration. Therefore, we tested different PEG concentrations to achieve the priming of *Coronilla varia* L. seeds and investigated this plant response to simulated water restrictions focusing on seed germination, seedling growth, osmotic regulatory substances content, and antioxidant enzymes’ activity. The key objectives of this study were to investigate the potential and significance of seed priming with PEG in mitigating water stress, as well as the mechanism by which priming enhances seed germination and seedling drought resistance in *Coronilla varia* L., thereby providing both a theoretical foundation and practical data for the rational use of priming agents in this forage cultivation.

## 2. Materials and methods

### 2.1. Materials

*Coronilla varia* L. seeds belonging to the “Emerald” variety were provided by Gansu Chuanglv Grass Industry Technology Co., Ltd. This variety is known for its strong resistance to adverse conditions and has been widely cultivated in Northwest China. The experiment was conducted from May to August 2022 at the College of Resources and Environmental Sciences, Gansu Agricultural University, in Lanzhou, China. Polyethylene glycol (PEG) 6000 was employed to generate increasing negative water potentials in the imbibition solutions.

### 2.2. Seed priming

Fully plump seeds of *Coronilla varia* L. of similar size were selected and disinfected using a 0.1% HgCl_2_ solution for 10 min. Subsequently, the seeds were rinsed with distilled water 3–4 times. Afterward, the surface moisture was removed with absorbent filter paper. Seed priming was achieved by placing the seeds into mesh bags and immersing them in 10%, 20%, and 30% PEG solutions, at a seed weight-to-solution ratio of 1:3 [(W(g):V(mL)]. The seeds were primed at 25°C in a germination chamber for 12 h. Then, they were rinsed, dried, spread on germination paper, and placed into a forced-air drying cabinet at 20°C until the total seed weight matched the pre-treatment weight. Unprimed seeds were used as controls. All seeds were stored at 4°C for subsequent experimentation [24].

### 2.3. Germination test of primed seeds

Unprimed seeds (labeled as CK) and primed seeds (labeled as 10%SP, 20%SP, and 30%SP) were placed in Petri dishes (diameter: 10 cm) with two layers of filter paper. Each treatment dish contained 60 seeds, with 6 repetitions per treatment. A volume of 8 mL of distilled water was added to each Petri dish. The covered plates were placed in a growth chamber under 8 h light/16 h darkness at 25°C, with a light intensity of 300 μmol·m^-2^·s^-1^ and 80% relative humidity [36,37]. Seed germination was monitored every 24 h; a 2 mm root protrusion through the seed coat was considered the germination standard. After 9 days, the seed germination rate and seedlings’ growth were measured.

### 2.4. Germination test under water stress

Two experimental factors were combined: water stress during germination simulated by different concentrations of PEG-6000 in the imbibition solution and previous seed priming (using 10% PEG). Primed and unprimed *Coronilla varia* L. seeds were used in a total of 8 treatments, with 6 repetitions each. Following the method described by Michel et al.[38], PEG6000 at 8%, 10%, and 12% was employed to generate water potentials during germination of −0.1, −0.15, and −0.2 MPa, respectively. In the control (CK), the PEG solution was replaced with distilled water. The experimental design and abbreviated treatment identifications are shown in Table 1.

**Table 1:** Experimental treatment.

Treatment	CK	CKP	G8	G8P	G10	G10P	G12	G12P
PEG during germination (%)	0	0	8	8	10	10	12	12
Previous seed treatment	unprimed	primed	unprimed	primed	unprimed	primed	unprimed	primed

A total of 60 seeds (primed or unprimed) were placed in each Petri dish (diameter: 10 cm) with two layers of filter paper, and 8 mL of the corresponding PEG-6000 solution was added to each dish. The dishes with the seeds were placed in a growth chamber under 8 h light/16 h darkness at 25°C, 300 μmol·m^-2^·s^-1^ light intensity, and 80% relative humidity [39]. The solutions were replaced every 2 days to maintain constant PEG concentrations. The germination rate was recorded daily for 9 days. Seed germination indices and seedling physiological indices were measured on the 10^th^ day.

### 2.5. Measurements

#### 2.5.1. Seed germination and seedling growth indices

The presence of a 2 mm root segment emerging from the seed coat was considered positive germination. Germination vigor (GV) was recorded on day 4, and germination percentage (GP) on day 9 [40]. Germination index (GI) [41] and vitality index (VI) [42] were calculated as detailed below. The time taken to achieve 50% of germination (T50) was calculated following the method of Agacka-Mołdoch [43].


$$GV=\left(\frac{\text{Number}\;\text{of}\;\text{seeds}\;\text{germinated}\;\text{during}\;\text{the}\;\text{first}\;4\;\text{days}}{\text{Total}\;\text{number}\;\text{of}\;\text{seeds}}\right)\times 100\text{\%}$$ (1)

$$GP=\left(\frac{\text{Total}\;\text{number}\;\text{of}\;\text{seeds}\;\text{germinated}\;\text{up}\;\text{to}\;\text{day}\;9}{\text{Total}\;\text{number}\;\text{of}\;\text{test}\;\text{seeds}}\right)\times 100\text{\%}$$ (2)

$$GI=\sum \left(\frac{Gt}{Dt}\right)$$ (3)

VI=GI×S (4)

Gt is the number of germinated seeds on day t and Dt is the day t.

S is the fresh weight of the seedlings (g).

Growth indices were measured on day 9. Ten random seedlings per treatment were selected to perform these measurements. A vernier caliper (accuracy: 0.01 mm) was used to measure the length from the root tip to the leaf end.

#### 2.5.2. Seedling physiological and biochemical indices

Proline (Pro) was determined as suggested by Bates [44] followed with some modifications. 0.1 g samples of plant material were placed in 10 mL test tubes. After adding 5 mL of 3% sulfosalicylic acid solution, the tubes were placed in a boiling water bath for 10 min and cooled to room temperature. Free proline in the supernatant was subsequently determined through the acid ninhydrin colorimetric method.

Soluble sugars (SS) was measured by the previously used method [45] with some modifications. 0.1 g samples of plant material were placed in test tubes. After adding 10 mL of distilled water and sealing with parafilm, the tubes were placed in a boiling water bath for 30 min. The extract was filtered and made up to 25 mL in a volumetric flask with distilled water. The anthrone colorimetric method was used to quantify SS.

Soluble protein (SP) was measured was measured by method of Asif Iqbal [45] with some modifications. 0.5 g of leaves were homogenized for 10 min after being pulverized in 6 mL of phosphate buffer solution (pH 7.0). The samples were placed in a water bath 25°C for 45 min and centrifuged at 4000× g for 5 min. The reaction mixture was composed of 5 mL, extract (1mL), and coomassie brilliant blue reagent (4mL). Finally, the values were recorded at 595 nm wavelength using distilled water as a blank control with the help of a spectrophotometer.

Membrane lipid peroxidation was determined by measuring malondialdehyde (MDA) content as described in Zhou et al. [46]. Seedling extracts were prepared from 1 g samples using 10 mL of l0% trichloroacetic acid (TCA) and a small amount of quartz sand to facilitate the grinding. The homogenate obtained was centrifuged at 4000 rpm for 10 min to obtain the supernatant fraction. Supernatant aliquots (1 mL) were mixed with 2.5 mL of 0.5% thiobarbituric acid (TBA) and heated at 100°C for 20 min. The absorbance was determined at 450, 532, and 600 nm.

Electrolyte leakage (EL) was evaluated to assess plasma membrane integrity using an electrical conductimeter (DDS-307A, INESA Analytical Instrument Co., Shanghai, P. R. China), according to Iqbal et al. [47]. The leaves were cut into small slices (5 mm long) and placed in test tubes containing 8 mL of distilled water. These tubes were incubated for 2 hours in a water bath (25°C), and the electrical conductivity was measured (initial EC, EC_1_). After autoclaving the samples at 121°C for 20 minutes, they were cooled down to 25°C, and the electrical conductivity was measured again (final EC, EC_2_). Then, the following formula was applied:

$$EL(\text{\%})=\frac{EC_{1}}{EC_{2}}\times 100$$ (5)

Seeding leaf samples (0.5 g) were extracted with 5 mL of phosphate buffer (50 mmol·L^-1^, pH 7.2) containing 100 μmol·L^-1^ EDTA, 1% PVP, 5 mmol·L^-1^ DTT), ground on an ice bath, and centrifuged at 12,000×*g*, 4°C, for 20 min. Enzyme activities were determined in the supernatant [48].

Catalase (CAT) activity was determined using UV spectrophotometry. The reaction mixture (1 mL) contained 0.4 mL of 100 mM potassium phosphate buffer (pH 7.0), 0.4 mL of 30% H_2_O_2_, and 0.2 mL of enzyme extract. The decline in the absorbance at 240 nm due to H_2_O_2_ consumption was recorded (*ε* = 39.4 mM^-1^cm^-1^) [49].

Peroxidase (POD) activity was measured colorimetrically using guaiacol. The concentration of POD correlates with guaiacol oxidation. The reaction mixture consisted of 100 μL enzyme extract, 50 mM phosphate buffer, 20 mM guaiacol, and 40 mMH_2_O_2_. Increases in the absorbance at 470 nm because of guaiacol oxidation was recorded for 1 min to determine the POD activity [50].

Superoxide dismutase (SOD) activity was determined using the nitroblue tetrazolium method. The functioning of SOD depends on the photochemical reduction of nitroblue tetrazolium (NBT) present in the reaction mixture at 560 nm. The reaction mixture contained 50 μM NBT, 50 μL enzyme extract, 1.3 μM riboflavin, 50 mM phosphate buffer, 75 nM EDTA, and 13 mM methionine. For photochemical oxidation, a 30 W light source was used for 15 minutes in a fluorescent chamber. The photochemical reduction of NBT produces formazone, which can be measured at 560 nm by UV-visible spectrophotometry [50].

#### 2.5.3. Statistical analysis

The IBM SPSS Statistics 26.0 (SPSS Inc., Chicago, IL, USA) software was used to conduct variance analysis (ANOVA), principal component analysis and spearman correlation test among the variables; significant differences were detected using the least significant difference (LSD) test at *P*-value<0.05. Mean values and standard errors (SE) are presented.

## 3 Results

### 3.1. Optimal PEG concentration for *Coronilla varia* L. seed priming

All priming treatments applied to *Coronilla varia* L. seeds led to an improved germination performance compared with unprimed seeds (CK) (Table 2); the best indices were observed when priming was conducted using 10% PEG. In that treatment, the germination percentage, germination vigor, germination index, and vitality index increased by 16.06%, 80.30%, 51.64%, and 72.36%, respectively, compared to the untreated control. Although the 30% PEG treatment enhanced some germination indices compared with unprimed seeds, the differences were not significant for other markers. The germination vigor, germination index, and vitality index of seeds treated with 30% PEG were 23.44%, 22.76%, and 32.10% reduced, respectively, compared to those recorded for 10% PEG, suggesting that too high PEG concentrations can be negative for seed germination.

**Table 2:** Effects of PEG priming on *Coronilla varia* L. seed germination and seedling growth.

Treatment	Germination percentage (%)	Germination vigor (%)	Germination index	Vitality index	T50	Seedling length (mm)	Seedling mass (mg)
CK	76.11±1.47 b	39.44±1.47c	12.49±0.18d	0.47±0.01b	5.33±0.33a	153.35±10.22a	37.27±1.36b
10%SP	88.33±2.55 a	71.11±1.47a	18.94±0.45a	0.81±0.01a	3.33±0.33c	160.92±1.95a	42.73±0.30a
20%SP	86.67±1.92 a	61.11±2.00b	16.76±0.08b	0.69±0.02a	4.00±0.00bc	152.56±5.35a	41.20±0.90ab
30%SP	81.67±3.47 ab	54.44±2.00b	14.63±0.09c	0.55±0.03b	4.33±0.33b	146.43±1.57a	37.43±1.98b

CK: Unprimed seeds; 10%SP, 20%SP, 30%SP: Seeds primed with 10%, 20%, and 30% PEG solutions, respectively. Different letters in the same column mean significant differences at *P*<0.05.

In addition, PEG priming shortened the T50 of the tested seeds, indicating an enhancement in seed germination uniformity. Specifically, the 10% PEG treatment accelerated germination by 2 days, resulting in more concentrated seed germination compared with other priming treatments. Though there were no significant differences in seedling length, the 10% PEG treatment resulted in a 12.78% increased mass compared to CK and a 12.40% increased mass compared to 30% PEG. No significant differences in fresh mass were observed when comparing the control with the seeds primed with 20% or 30% PEG. This suggests that under normal watering conditions, only a low PEG concentration effectively promotes seed germination and seedling growth.

### 3.2. Seed priming effects on germination and seedling growth in *Coronilla varia* L. subjected to water stress

**Fig 1:**
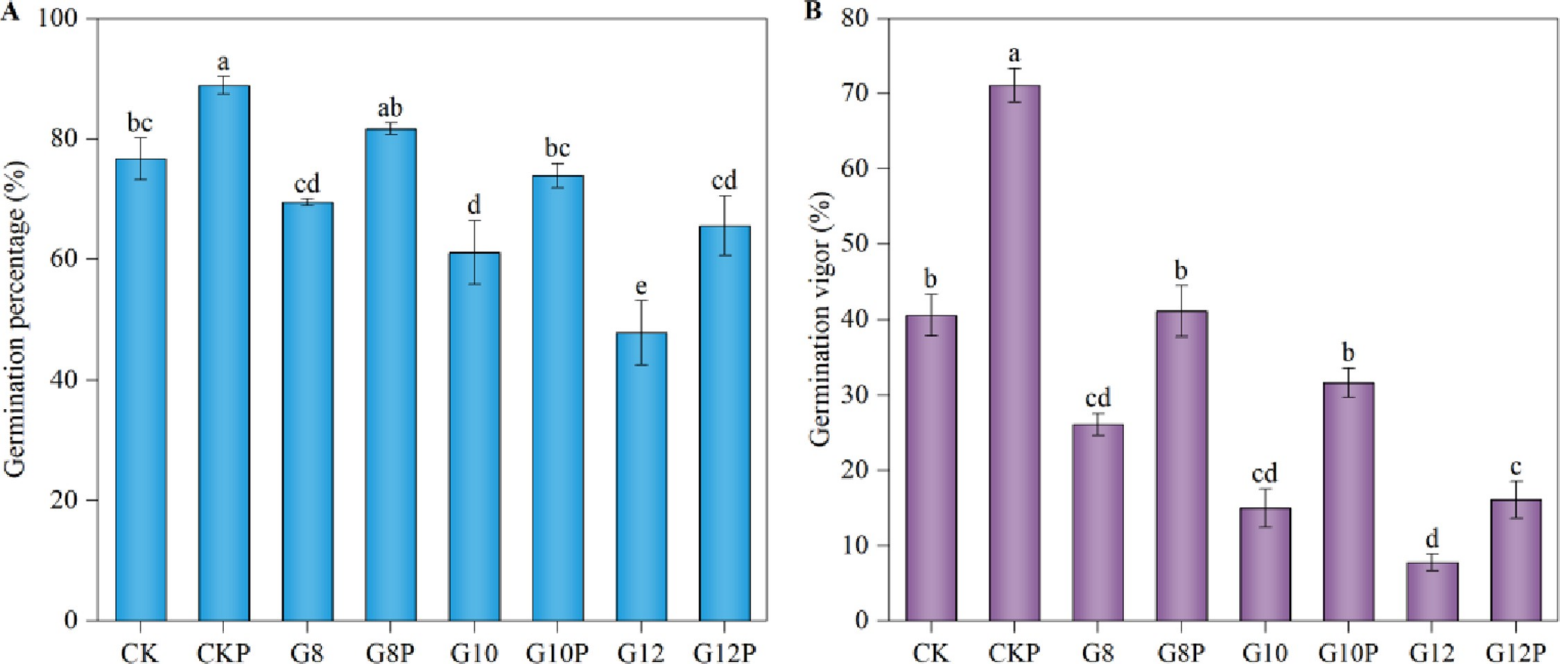
Effect of seeds priming with 10% PEG on germination percentage (A) and germination vigor (B) of *Coronilla varia* L. under varying water stress conditions. Different lowercase letters indicate significant differences (*P*<0.05). Values are means ± standard errors. Treatment abbreviations correspond to those detailed in the experimental design summary (Table 1).

It may be observed in Fig 1 that reduced water potentials due to PEG incorporation into the imbibition medium significantly diminished both the germination percentage (Fig 1A) and germination vigor (Fig 1B) of *Coronilla varia* L. seeds in a dose-dependent manner. Thus, the germination percentage and vigor of G8, G10, and G12 treatments were reduced by 9.43%, 20.29%, and 37.68%, and by 35.63%, 63.02%, and 80.82%, respectively, compared to non-stressed seeds (CK). However, the previous seed priming (using 10% PEG) had positive effects and increased both the germination percentage and vigor of these small seeds. Under water stress, the germination percentage and vigor were increased respectively by 81.67% and 41.11% with G8P 73.89% and 31.67% with G10P treatment when compared to G8 and G10 treatments, yet they were not significantly different from CK. Even at a water potential as low as −0.2 MPa (G12 and G12P), seed priming had a positive effect on *Coronilla varia* germination. In this way, we proved that seed priming can promote the germination of this species under both sufficient water supply and low or medium water restrictions, mitigating the inhibitory effect of this stress on seed germination.

**Fig 2:**
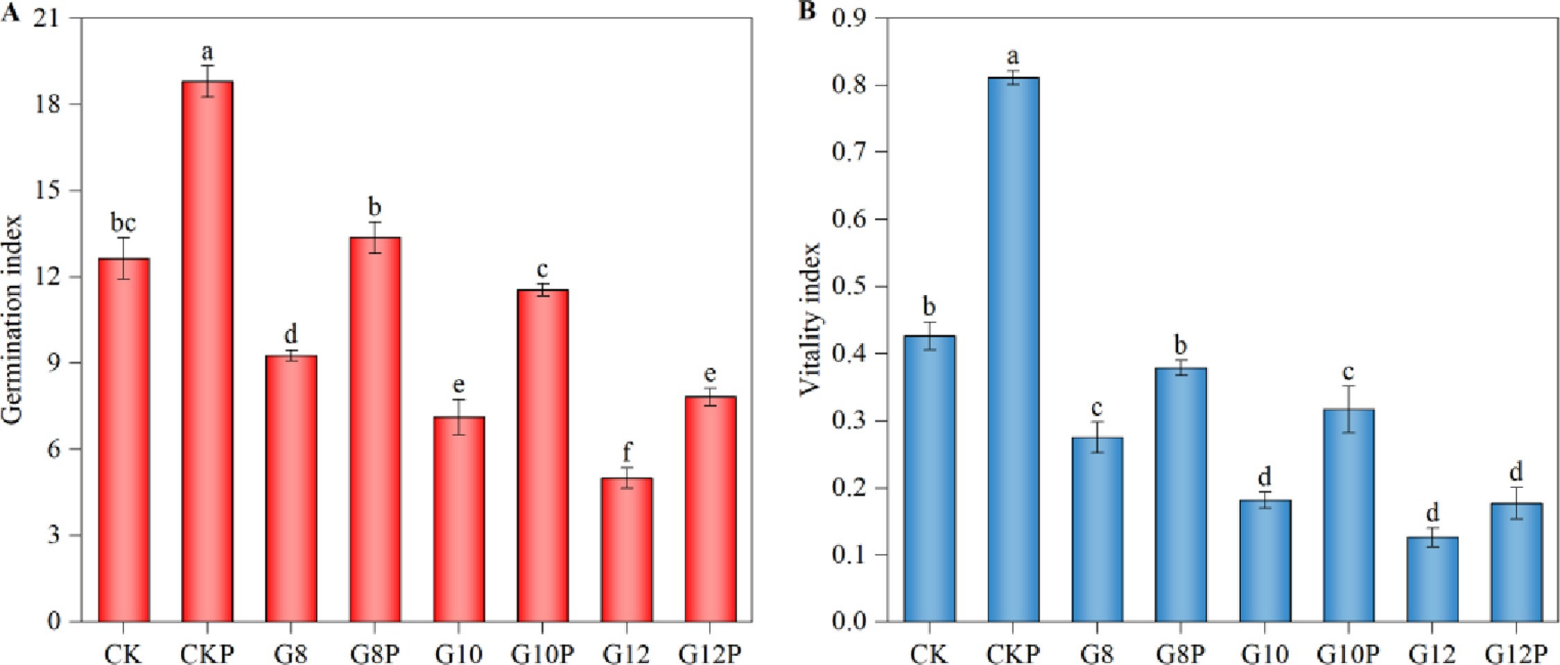
Effect of seeds priming with 10% PEG on germination index (A) and vitality index (B) of *Coronilla varia* L. under varying water stress conditions. Different lowercase letters indicate significant differences (P<0.05). Values are means of replicates ± standard errors. Treatment abbreviations correspond to those detailed in the experimental design summary (Table 1).

Decreased water potentials caused by increasing PEG amounts also reduced the germination index (Fig 2A) and vitality index (Fig 2B) of *Coronilla varia* L. Under 8% PEG-induced water stress, the germination index and vitality index of the primed seeds (G8P) were similar to those of the unstressed and unprimed seeds (CK). However, under 12% PEG-induced water deficiency, both the germination index and the vitality index in the primed treatment decreased significantly compared to CK, and the germination index but not the vitality index was significantly improved due to priming. This suggests that 10% PEG priming can enhance seed vitality if faced with water limitations, but its effectiveness diminishes if the limitation is very strong.

**Fig 3:**
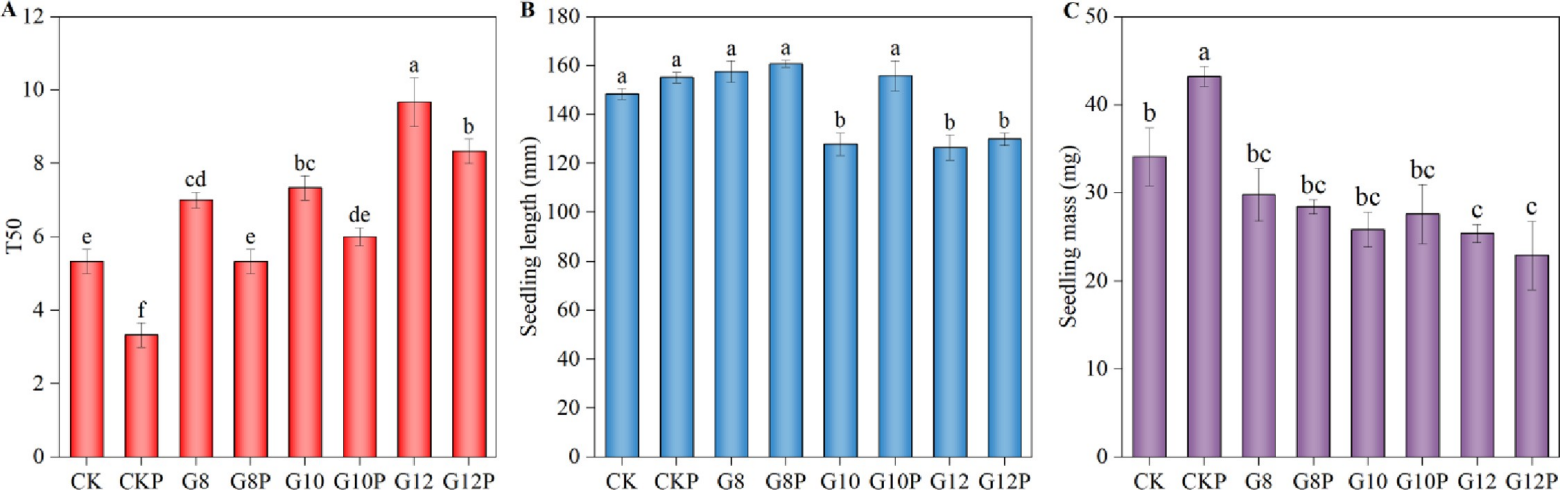
Effect of seeds priming with 10% PEG on T50 (A), seedling length (B), and seedling mass (C) of *Coronilla varia* L. under varying water stress conditions. Different lowercase letters indicate significant differences (*P*<0.05). Values are means of replicates ± standard errors. Treatment abbreviations correspond to those detailed in the experimental design summary (Table 1).

Another physiological parameter altered due to water limitation was the time to achieve 50% germination (T50) (Fig 3A). Priming (10% PEG) shortened this time significantly in non-water restricted seeds as well as in seeds subjected to negative water potentials. G8 and G10 treatments resulted in a 2-day increase in T50 compared to CK and G12 treatment in a 4-day increase. The priming of the seeds with 10% PEG reduced the T50 significantly across all levels of water deficits, with an average reduction of 1.4 days. This implies that priming improved seed germination uniformity.

A mild water deficit achieved by adding 8% PEG in the imbibition solution did not significantly affect the seedling growth (Fig 3B) or seedling mass (Fig 3C), but the addition of 12% PEG significantly reduced both parameters compared to CK (by 21.88 mm and 8.7 mg, respectively), with no significant difference when compared with the response of primed seeds exposed to the same condition.

### 3.3. Seed priming effects on osmoregulatory substances accumulation in *Coronilla varia* L. subjected to water stress

**Fig 4:**
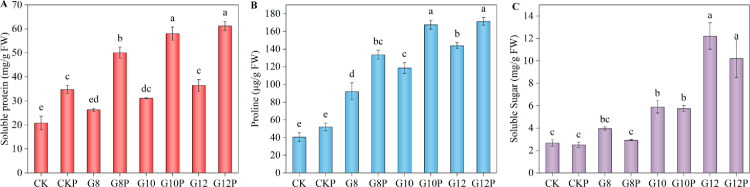
Effect of seeds priming with 10% PEG on soluble protein (A), proline (B), and soluble sugar (C) content in *Coronilla varia* L. seedlings grown under varying water stress conditions. Different lowercase letters indicate significant differences (*P*<0.05). Values are means of replicates ± standard errors. Treatment abbreviations correspond to those detailed in the experimental design summary (Table 1).

Both seed priming and water limitation significantly elevated the soluble protein content in *Coronilla varia* L. tissues (Fig 4A). Thus, unprimed non-stressed seedlings (CK) had 40.06% less soluble protein than non-stressed primed (CKP) seedlings. From a basal value of 20.84 mg soluble protein/g FW (CK), a maximum of 61.21 mg soluble protein/g FW was recorded for G12P treatment. The maximum value for unprimed seeds was 58.03 mg soluble protein /g FW at G12.

The content of the osmolyte proline did not vary due to seed priming under normal water conditions but significantly increased when PEG was added to the imbibition solution (Fig 4B). Due to water limitation, the proline levels in G8, G10, and G12 treatments increased by 126.83%, 191.61%, and 253.96%, respectively, compared to CK, and seed priming (10% PEG) resulted even in higher values.

The soluble sugar content in *Coronilla varia* seedlings remained relatively stable between 2.52 and 3.96 mg/g FW if no water restriction was imposed or a small amount of PEG (8%) was incorporated in the imbibition solution to induce a mild water limitation, regardless of priming (Fig 4C). However, the soluble sugar content increased notably and proportionally under more pronounced water restrictions, reaching about 6.00 mg/g FW and 10.00–12.00 mg/g FW when 10% and 12% PEG, respectively, was added to the imbibition medium, with no significant differences between primed and unprimed seedlings.

### 3.4. Seed priming effects on cell membrane damage in *Coronilla varia* L. subjected to water stress

**Fig 5:**
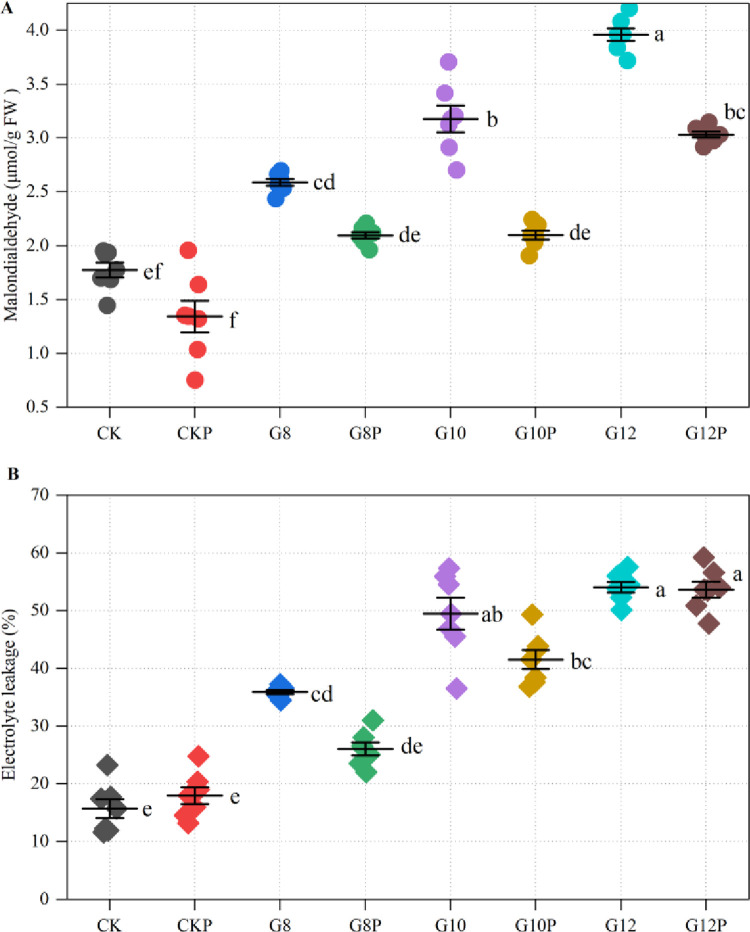
Effect of seeds priming with 10% PEG on MDA content (A) and electrolyte leakage (B) in *Coronilla varia* L. seedlings grown under varying water stress conditions. Different lowercase letters indicate significant differences (*P*<0.05). Values are means of replicates ± standard errors. Treatment abbreviations correspond to those detailed in the experimental design summary (Table 1).

We measured MDA content in *Coronilla varia* L. tissues to detect if water limitation caused lipid peroxidation due to excessive ROS levels. As shown in Fig 5A (Fig 5A), seed priming did not alter basal MDA levels in non-stressed seedlings, and water limitation imposed by PEG addition to the imbibition solution led to enhanced MDA contents in a dose-dependent manner. These rises, however, were less pronounced in primed seedlings compared with their unprimed counterparts, revealing a mitigating effect regarding oxidative stress. The mitigating effects were significant under moderate (10% PEG) and strong (12% PEG) water restriction. These findings suggest that *Coronilla varia* seed priming can alleviate the lipid peroxidation induced by moderate to high water limitations during this initial developmental stage.

Because the oxidative damage to lipidic cell membrane components is expected to affect cell membrane permeability, leading to increased diffusion of solutes, we also determined electrolyte leakage (Fig 5B). It may be observed that seedlings developed from unprimed (CK) and primed (CKP) *Coronilla varia* L. seeds had similar electrolyte leakage levels under water sufficient supply. However, water stress gradually enhanced the electrolyte leakage. Thus, under G8, G10, and G12 treatments, the electrolyte leakage was increased by 128.53%, 215.10%, and 244.33%, respectively, compared to CK, being significant in the last two. The seed priming treatment mitigated these electrolyte leakage increases under the mild (8% PEG) and the moderate (10% PEG) water stress but not at the most severe water deficit (12% PEG-induced).

### 3.5. Antioxidant enzyme activities in the leaves of *Coronilla varia* L. subjected to water stress

**Fig 6:**
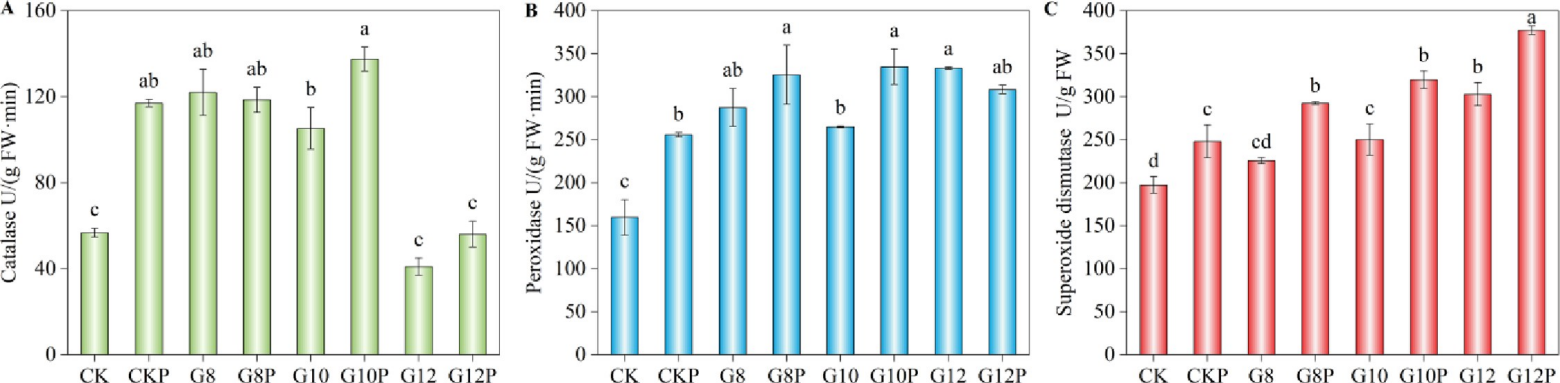
Effect of seeds priming with 10% PEG on CAT (A), POD (B), and SOD (C) activity in *Coronilla varia* L. seedlings grown under varying water stress conditions. Different lowercase letters indicate significant differences (*P*<0.05). Values are means of replicates ± standard errors. Treatment abbreviations correspond to those detailed in the experimental design summary (Table 1).

We determined the activity of three key antioxidant enzymes, catalase (CAT), peroxidase (POD), and superoxide dismutase (SOD), in *Coronilla varia* L. leaf tissue. With increasing PEG concentrations in the imbibition solution, CAT activity first increased and then declined (Fig 6A). The highest CAT activity among unprimed units was 122.02 U/g FW·min in G8 seedlings, a value significantly higher than that of the control (CK). Subsequently, CAT activity in unprimed seedlings started to decrease, reaching a minimum of 41.04 U/g FW·min in G12 seedlings. Seed priming enhanced CAT activity in non-stressed seedlings (CKP) and moderately water-stressed seedlings (G10P), reaching 137.50 U/g FW·min, a value 30.60% higher than the unprimed G10. However, under a strong water deficit, CAT activity was not significantly different from the control (CK) neither in unprimed (G12) nor primed (G12P) seedlings.

POD showed a relatively similar pattern (Fig 6B), but unlike CAT, at the strongest water restriction (12% PEG), its activity was significantly higher than in the unstressed unprimed control (CK). POD activity in CKP was 60.18% higher than in CK, and G10P activity was 26.39% higher than G10 activity; both differences reached significant levels. No significant difference was observed between G12 and G12P treatments.

Finally, both water stress and seed priming tended to stimulate SOD activity sustainedly (Fig 6C). Thus, *Coronilla varia* leaves developed from primed seeds showed the maximum POD activity, 377.31 U/g FW, when subjected to 12% PEG (G12P), being this value significantly higher than all others. The minimum SOD activity was of about 200 U/g FW in CK. Seedlings from primed seeds displayed SOD enzyme activities 24–30% higher compared to unprimed ones.

### 3.6. Relationship among diverse parameters: Two-way ANOVA, principal component analysis and Pearson correlation heat map

A two-factor ANOVA was required to examine the interaction effects between factors. The two experimental factors were water stress, simulated by different concentrations of PEG-6000 in the imbibition solution, and previous seed priming using 10% PEG (Table 3). It may be noticed that WS, as a sole factor, significantly affected all physiochemical parameters. PT as a sole factor and the interaction WS x PT had significant impact on GV, GI, VI, SL, SP, Pro, CAT and POD, but no significant impact on other parameters.

**Table 3:** Physiochemical parameters of *Coronilla varia* L. seeds measured after seed priming using 10% PEG and analyzed through ANOVA (with mean square and *p* values).

Variation Source	df	GP	GV	GI	VI	T50
Water Stress (WS)	3	0.075^***^ (0.000)	0.209^***^ (0.000)	91.565^***^ (0.000)	0.243^***^ (0.000)	22.153^***^ (0.000)
Priming Treatment (PT)	1	0.113^***^ (0.000)	0.187^***^ (0.000)	114.917^***^ (0.000)	0.171^***^ (0.000)	15.042^***^ (0.000)
WS × PT	3	0.001^ns^ (0.831)	0.013 ^**^ (0.002)	2.860^*^ (0.021)	0.033^***^ (0.000)	0.153 ^ns^ (0.750)
Error	16	0.004	0.002	0.663	0.001	0.375
Variation Source	df	SL	SM	SP	Pro	SS
Water Stress (WS)	3	1074.397^***^ (0.000)	0.000^***^ (0.000)	502.067^***^ (0.000)	14637.304^***^ (0.000)	89.982^***^ (0.000)
Priming Treatment (PT)	1	642.218^**^ (0.002) ^*^	0.000 ^ns^ (0.346)	2992.371^***^ (0.000)	6254.873^***^ (0.000)	4.220 ^ns^ (0.143)
WS × PT	3	210.391^*^ (0.018)	0.000 ^ns^ (0.146)	49.687^*^ (0.027)	412.119^*^ (0.018)	1.169 ^ns^ (0.590)
Error	16	46.711	0.000	12.439	92.265	1.781
Variation Source	df	MDA	EL	CAT	POD	SOD
Water Stress (WS)	3	3.847^***^ (0.000)	0.160^***^ (0.000)	7104.103^***^ (0.000)	15861.117^***^ (0.000)	14649.996^***^ (0.000)
Priming Treatment (PT)	1	3.224^***^ (0.000)	0.010 ^ns^ (0.145)	4060.913^***^ (0.000)	12062.235^**^ (0.003)	25683.953^***^ (0.000)
WS × PT	3	0.154 ^ns^ (0.259)	0.005 ^ns^ (0.322)	1097.216^**^ (0.001)	4074.674^*^ (0.022)	156.735 ^ns^ (0.772)
Error	16	0.104	0.004	124.552	956.000	417.637

df, degree of freedom; ns, non-significant; *, **, and *** = significant at 0.05, 0.01, and 0.001 levels, respectively; GP, germination percentage; GV, germination vigor; GI, germination index; VI, vitality index; T50, time to 50% germination; SL, seedling length; SM, seedling mass; SP, soluble protein, Pro, proline; SS, soluble sugar; MDA, malondialdehyde content; EL, electrolyte leakage; CAT, catalase; POD, peroxidase; SOD, superoxide dismutase.

**Fig 7:**
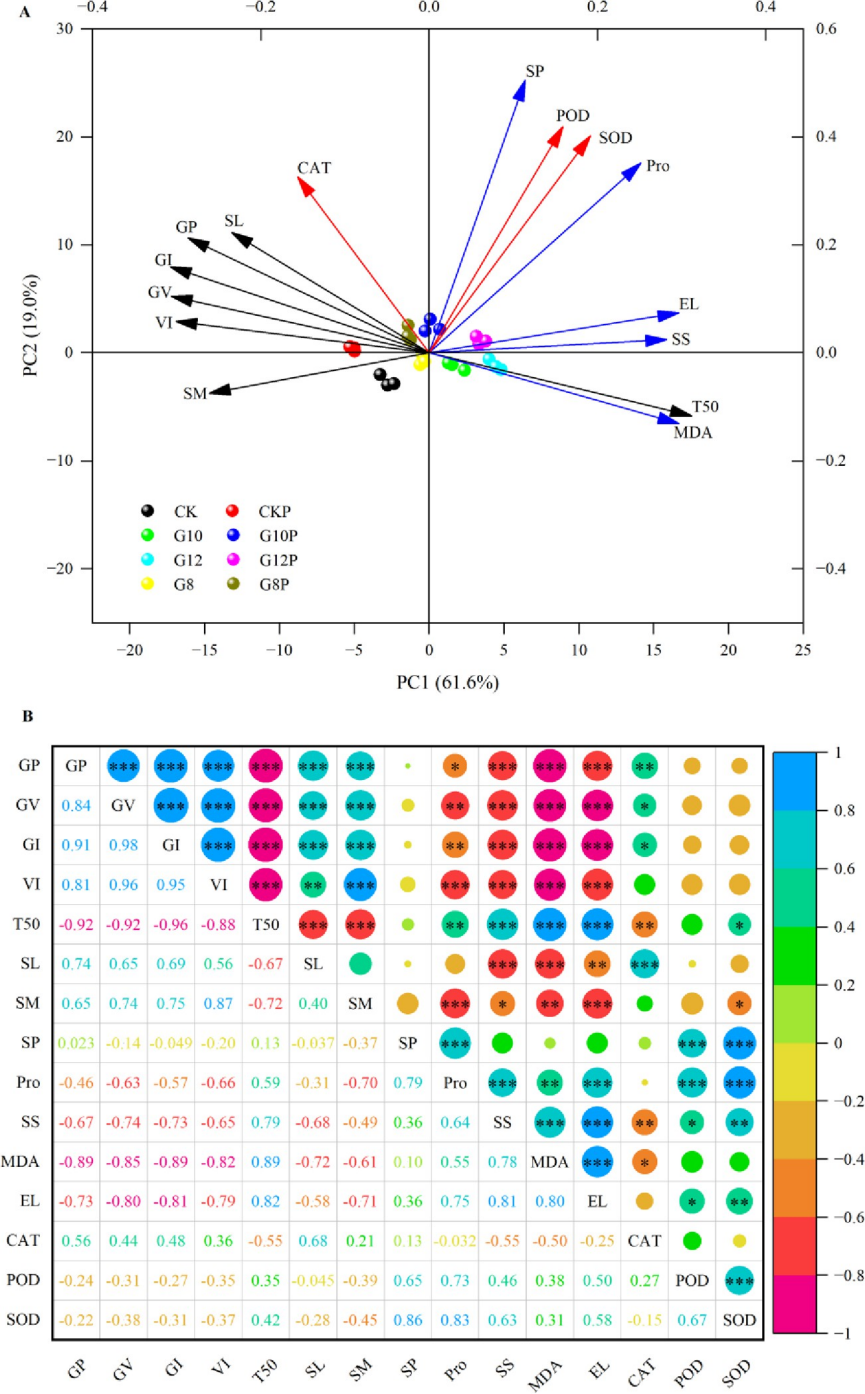
Principal component analysis (A) and Pearson correlation heat map (B) depicting the relationships among diverse biochemical and physiologic parameters of *Coronilla varia* L. subjected to seed priming and water stress. GP, germination percentage; GV, germination vigor; GI, germination index; VI, vitality index; T50, time to 50% germination; SL, seedling length; SM, seedling mass; SP, soluble protein, Pro, proline; SS, soluble sugar; MDA, malondialdehyde content; EL, electrolyte leakage; CAT, catalase; POD, peroxidase; SOD, superoxide dismutase; *, **, and *** = significant at 0.05, 0.01, and 0.001 levels, respectively.

Principal component analysis (PCA) was conducted to explain the contribution of diverse biochemical and physiologic indexes to the total variability observed in this analysis of *Coronilla varia* L. response to seed priming and water limitation (Fig 7A). The first principal component was composed of GP, GV, GI, VI, T50, SL, SM, SS, MDA, and EL, and its contribution rate reached 61.6%. The second principal component was composed of SP, Pro, CAT, POD, and SOD, and its contribution rate reached 19.0%. Through the Pearson correlation heat map (Fig 7B), we found that GP was significantly positively correlated with GE, GI, VI, SL, and SM and negatively correlated with T50, indicating that better germination indexes can shorten the time taken for 50% germination, improve germination uniformity, and promote seedling growth. GP was significantly negatively correlated with MDA and EL, implying that cell membrane oxidative damage and disruption resulted in impaired germination. GP was significantly positively correlated with CAT activity, suggesting that high CAT activity may be necessary for efficient ROS detoxification and improved seed germination.

## 4. Discussion

Drought stress can have a detrimental impact on plant morphology, physiology, crop yields, biomass, and global food security. Through seed osmotic priming, plants may reach a specific physiological state in which the rate and amount of water uptake before germination are controlled [51]. This condition ensures increased and uniform germination by reducing the imbibition time, increasing the activation of pre-germinative enzymes, facilitating the restoration of the seed membrane system, reducing nutrient leakage, increasing metabolite production, repairing damaged DNA, and regulating osmosis [10–15]. Over the past few years, seed priming has emerged as a promising strategy in modern biotic and abiotic stress management [11].

Mouradi et al. found that after priming *Medicago sativa* L. seeds with 20% polyethylene glycol (PEG6000) for 24 h, both germination percentage and seedling growth rate were higher compared with untreated seeds. Their results suggest that priming stimulates the metabolic activity of *Medicago sativa* seeds and promotes seed germination [52]. In our study, polyethylene glycol (PEG6000) served as an ideal osmotic priming agent, and moderate PEG priming significantly improved seed germination percentage, germination vigor, germination index, vitality index, and seed germination uniformity (Table 2) in *Coronilla varia* L. The optimal PEG concentration for the priming of this leguminous forage was 10%. Similar results were reported for *Medicago sativa* [52], *Oryza sativa* L. [53], and *Sorghum bicolor* L. [25]. It has been indicated that primed seeds acquire the potential to rapidly imbibe and revive the seed metabolism, thus enhancing the germination rate [15]. Some studies have shown that PEG priming can enhance growth, leaf area, and plant height in *Medicago sativa* L. seedlings [54]. In our study, priming did not significantly affect seedling length in *Coronilla varia* L., and only 10% PEG priming enhanced seedling mass. This suggests differential effects of osmotic priming on different plant species, supporting the notion that priming is influenced by the biological characteristics of the plant species and variety and that initial seed mass is a relevant feature regarding seed priming effects [13].

The successful establishment of seedlings requires rapid and uniform emergence, particularly when environmental conditions are limiting. The susceptibility of young seedlings to a range of abiotic and biotic stressors has been extensively demonstrated [55]. Water availability is a major limiting factor during seed germination and seedling growth. Water deficiency reduces germination vigor, germination percentage, germination index, and vitality index and ultimately suppresses growth and productivity [3]. Seed priming has been shown to enhance seed germination and post-emergence drought resistance in many plant species [11]. Osmotic priming reduced the water potential required for seed germination of *Satureja montana* L. [56] and *Triticum aestivum* L. [57], promoting rapid seed germination and enhancing drought tolerance during this fundamental process.

In our study, all germination indices of *Coronilla varia* L. significantly decreased under water stress, but 10% PEG priming mitigated these adverse effects under mild and moderate water restrictions, in line with previous findings. Studies on primed *Cicer arietinum* L. [58] and *Zinnia elegans* Jacq. [59] seeds revealed effects on emergence uniformity (T50), but the optimal concentration of the priming agents varied, suggesting heterogeneous adaptive mechanisms regarding priming agents and concentrations in different plants. We found that germination and growth indices of primed *Coronilla varia* L. diminished under high water stress (12% PEG), but they still surpassed those of the unprimed seedlings. This might be attributed to an enhanced vigor in primed seeds under water deficit. However, the stimulated adaptability of *Coronilla varia* L. to the external environment via priming treatment remains limited. The principal component analysis (PCA) shown in Fig 7A indicates two key components, PC1 and PC2, contributing to 61.6% and 19.0% of the variance, with a cumulative participation of 80.6%. The Spearman correlation heatmap in Fig 7B shows highly significant correlations among the studied variables. All the studied attributes were strongly correlated with the seed germination traits of *Coronilla varia* L. in the present study.

To adapt to drought conditions, plants accumulate significant amounts of osmotic regulatory substances such as proline and soluble proteins. This mechanism elevates intracellular osmotic pressure, maintains cellular turgor, and prevents excessive cellular dehydration. It also stabilizes cellular structures and maintains normal metabolism within the cells [6]. We found that both water deficit and priming treatment significantly increased proline and soluble protein contents in *Coronilla varia* seedlings compared with unprimed seedlings. Proline and soluble protein play a critical part in the mechanism of osmotic adjustment in many crops under severely stressed conditions, and a rise in their levels in plants during drought stress is thought to be a sign of drought stress resistance[60]. Increased protein synthesis was reported in the root tips of *Brassica oleracea* L. after osmotic priming [61], and Patane et al. [62] communicated similar results for *Sorghum bicolor*. We corroborated that *Coronilla varia* L. can accumulate osmotic regulatory substances to cope with insufficient water levels, and PEG priming can further promote the production of these substances, thus maintaining cellular osmotic balance and enhancing drought resistance. These osmolytes gathering can help the crop survive short periods of drought and recover from such stress, but long-term exposure to water-deficit conditions, as predicted by future climate change, will require not only the use of growth regulators but also biotechnological and breeding efforts[63]. Mukasa et al. [64] found that rapeseed priming increased α-amylase activity within seed endosperm, facilitating starch hydrolysis and increasing sugar supply. In our study, as the intensity of water stress increased, soluble sugar content in *Coronilla varia* L. seedlings increased, but seed priming did not result in additional rises. Further research is needed to understand how seed priming regulates plant sugar and energy metabolism in this plant species.

In plant seeds, several enzymes are involved in the continuous renewal and repair of cell membranes while clearing toxic substances produced from the self-oxidation of unsaturated fatty acids in the membrane phospholipids. When plants face adverse conditions, the activity of these antioxidant enzymes usually decreases, causing ROS to accumulate rapidly within the cells, causing enhanced lipid peroxidation and resulting in increased MDA levels and electrolyte leakage [8,52]. Thus, MDA content reflects the degree of lipid peroxidation, while electrolyte leakage indicates the extent of the cell membrane damage [46,48]. Both markers increased due to water restrictions in *Coronilla varia* L. Similar results have been reported for *Solanum lycopersicum* L.[28] and *Glycine max* L.[4,65]. Interestingly, the priming treatment of *Coronilla varia* L. seeds resulted in less oxidative damage under water restriction, reflecting the potential of this mechanism to alleviate the oxidative cascade triggered under water shortage. Nonetheless, under severe water deficiency (12% PEG), the protective effect of priming on leaf membrane structure diminishes so that intense electrolyte leakage cannot be prevented.

Within their specific tolerance range, plants can trigger a series of protective mechanisms to keep ROS at non-toxic levels; this includes the activation of the antioxidant enzyme system [15]. By targeting various ROS, including hydrogen peroxide (H_2_O_2_), hydroxyl radicals (-OH), superoxide radicals (O^2−^), and singlet oxygen (-O^2^), these enzymes are essential to cope with adverse environmental conditions [66,67]. It has been recognized that priming itself imposes stress on the seeds, inducing cellular defenses [11,19]. We found that primed (10% PEG) *Coronilla varia* L. seedlings had enhanced antioxidant enzyme activities under normal water supply, but most of these activities were even higher under water restriction, improving the plant’s ability to eliminate free radicals through better internal defense mechanisms [68]. This indicate that seed priming assists plants in developing various antioxidant enzyme systems to survive under oxidative stress caused by unfavourable environmental conditions. Other studies have reported comparable results. For instance, the antioxidant enzyme activities in *Spinacia oleracea* L. seeds after −0.6 MPa PEG priming were higher than those in unprimed seeds, and this could alleviate the inhibition of seed germination due to drought stress [68,69]. Also, PEG priming of *Psophocarpus tetragonolobus* L. [70] and *Sorghum bicolor* L. [25,71] boosted the activities of protective enzymes like CAT, POD, and SOD. In our research, antioxidant enzyme activities declined upon exposure to water stress. We detected significantly enhanced SOD, CAT, and POD activities in primed seedlings. Seed priming with PEG may have served as a catalyst that enhanced the activities of these enzymes and ultimately reduced MDA and H_2_O_2_ levels. These pieces of evidence collectively suggest that under water stress, PEG priming contributes to the maintenance of lower ROS levels by enhancing antioxidant enzyme activity.

Some limitations of this research should be mentioned. First, a single priming time (12 h) was tested; different priming periods might result in different outcomes. Second, all experiments were performed in laboratory conditions. Further research in actual field production settings is necessary to determine whether the positive effects of seed priming could result in superior germination rates and plant stands. Under current *Coronilla varia* sowing practices in China, the usual approach to ensure adequate seedling stands is by increasing seed density. This directly pushes up sowing costs. Our results indicate that seed priming using an appropriate PEG concentration can effectively promote germination, enhance germination vigor and percentage, and stimulate seedling growth. Summing up, our study provides theoretical and practical guidance to improve *Coronilla varia* L. cultivation in a cost-effective manner.

## 5. Conclusions

Water stress adversely affects *Coronilla varia* L. seed germination and plant growth and generates oxidative imbalances, resulting in cell membrane damage and increased cell permeability. Seed priming with 10% PEG moderately mitigated the growth-inhibiting effects of water stress in *Coronilla varia* L. seedlings by enhancing the activities of antioxidant enzymes, thereby contributing to the elimination of ROS and mitigation of cell membrane damage. Additionally, seed priming increased the concentration of osmoregulatory substances and enhanced the ability of *Coronilla varia* L. cells to regulate osmotic imbalances.

Thus, priming the seeds with 10% PEG can promote uniform and rapid *Coronilla varia* L. germination, resulting in earlier seedling growth and increased seedling tolerance to water deficit.

## Supporting information

S1 Data All seed priming data on germination and seedling physiological traits of *Coronilla varia* L. under water stress. (XLSX)
